# Pathogenic priming likely contributes to serious and critical illness and mortality in COVID-19 via autoimmunity

**DOI:** 10.1016/j.jtauto.2020.100051

**Published:** 2020-04-09

**Authors:** James Lyons-Weiler

**Affiliations:** The Institute for Pure and Applied Knowledge, USA

**Keywords:** SARS-CoV-2, COVID-19, Autoimmunity, Pathogenic priming, Immune Enhancement

## Abstract

Homology between human and viral proteins is an established factor in viral- or vaccine-induced autoimmunity. Failure of SARS and MERS vaccines in animal trials involved pathogenesis consistent with an immunological priming that could involve autoimmunity in lung tissues due to previous exposure to the SARS and MERS spike protein. Exposure pathogenesis to SARS-CoV-2 in COVID-19 likely will lead to similar outcomes. Immunogenic peptides in viruses or bacteria that match human proteins are good candidates for pathogenic priming peptides (similar to the more diffuse idea of “immune enhancement”). Here I provide an assessment of potential for human pathogenesis via autoimmunity via exposure, via infection or injection. SAR-CoV-2 spike proteins, and all other SARS-CoV-2 proteins, immunogenic epitopes in each SARS-CoV-2 protein were compared to human proteins in search of high local homologous matching. Only one immunogenic epitope in a SARS-CoV-2 had no homology to human proteins. If all of the parts of the epitopes that are homologous to human proteins are excluded from consideration due to risk of pathogenic priming, the remaining immunogenic parts of the epitopes may be still immunogenic and remain as potentially viable candidates for vaccine development. Mapping of the genes encoding human protein matches to pathways point to targets that could explain the observed presentation of symptoms in COVID-19 disease. It also strongly points to a large number of opportunities for expected disturbances in the immune system itself, targeting elements of MHC Class I and Class II antigen presentation, PD-1 signaling, cross-presentation of soluble exogenous antigens and the ER-Phagosome pathway. Translational consequences of these findings are explored.

## Highlights

• All SARS-CoV-2 immunogenic epitopes have similarity to human proteins except one.
• Roughly one-third of the potentially targeted human proteins (putative autoantigens) are key players in the adaptive immune system.
• The list of viral/human protein matches provides clues on which epitopes or parts of epitopes might be involved in the immunopathogenesis of COVID-19 disease from SARS-CoV-2 infection.
• It also indicates which epitopes might be responsible for autoimmunological pathogenic priming due to prior infection or following exposure to SARS-CoV-2 or relatives following vaccination.
• These epitopes should be excluded from vaccines under development to minimize autoimmunity due to risk of pathogenic priming.

## 1 Introduction

Autopsies of Chinese citizens who have died from COVID-19 following SARS-CoV-19 infection show evidence of interstitial changes, suggesting the development of pulmonary fibrosis [1]. This suggests, at least partly, an autoimmunology basis of the pathogenesis of COVID-19. A number of causal bases of autoimmunity from exposure to viral epitopes is well established, whether the route of administration be by exposure via infection or vaccination. Systemic respiratory viral infections can have numerous serious health effects, including dyspnea, hypoxemia, dysuria, meningitis, low blood pressure, shock and death. Sequalae of viral infections can include various short-term and sometimes permanent effects on the central nervous system, via direct injury to CNS tissue due to viral replication (viral neuropathology) or due to the host immune system learning to attack CNS tissue (viral neuroimmunopathology). The public health consequences from some viruses can include impaired immunity, encephalitis, and long-term neurological diseases such as demyelinating disorders and relapsing events such as those seen in multiple sclerosis (MS).

Both genetic and environmental factors are thought to contribute both to the severity of viral infections, and in determining who will develop autoimmune conditions. Various specific mechanisms (etiologies) of autoimmunity are recognized as plausible in viral induction of autoimmunity, including epitope spreading, molecular mimicry, cryptic antigen, and bystander activation, each a plausible etiological mechanism responsible for activating self-reactive (SR) immune cells.

Mortality in SARS-CoV-2 infection from COVID-19 is highly age-dependent, with older patient having the highest probability of death. The etiology of the age-specific mortality seen in COVID-19 is largely unknown. SARS-CoV and SARS-CoV-2 coronaviruses targets the epithelial cells of the respiratory tract, resulting in diffuse alveolar damage. Other tissues are obviously also potential targets for viral immunopathology, including mucosal cells of the intestine, epithelial cells of the kidneys, brain cells (neurons), and cellular components of the immune system. In severe cases of SARS, and likely in SARS-CoV-2, this viral targeting leads acutely to pneumonia. Exposure to other viral and ambient antigens are known causal factors in chronic autoimmune diseases of the airways including asthma. The role of autoimmunity in enhancing the severity of secondary exposures following prior infection or vaccinations has been given little consideration.

Among coronaviruses, the spike surface glycoprotein is known to play a role in neuroimmunopathology. However, the SARS-CoV-2 virus has numerous other proteins and polyproteins, each which may serve as an antigen source during infection leading to autoimmunity. the immune system presents those proteins, like all human proteins, as “normal”. T-cells are trained to recognize a normal protein shape in the thymus. Biomimicry between or among proteins from pathogens (infection or injection) can conflate the signaling by creating a population of memory B-cells, especially if the reaction switches from a Th-1 toward a Th-2 response.

In SARS, a type of “priming” of the immune system was observed during animal studies of SARS spike protein-based vaccines leading to increased morbidity and mortality in vaccinated animals who were subsequently exposed to wild SARS virus. The problem, highlighted in two studies, became obvious following post-vaccination challenge with the SARS virus [2]. found that recombinant SARS spike-protein-based vaccines not only failed to provide protection from SARS-CoV infection, but also that the mice experienced increased immunopathology with eosinophilic infiltrates in their lungs. Similarly [3], found that ferrets previously vaccinated against SARS-CoV also developed a strong inflammatory response in liver tissue (hepatitis). Both studies suspected a “cellular immune response”.

These types of unfortunate outcomes are sometimes referred to as “immune enhancement”; however, this nearly euphemistic phrase fails to convey the increased risk of illness and death due to prior exposure to the SARS spike protein. For this reason, I refer to the concept as “pathogen priming”; the peptides with pathogenic potential therefore are referred to as “putative pathogenic priming peptides”.

In this study, I present the likely human epitopic targets of biomimicry-induced autoimmunological components of morbidity and mortality caused by SARS-CoV-2 infection. This is achieved via bioinformatics analysis of the homology between highly immunogenic SARS-CoV-2 epitopes and human proteins to promote comprehension of the etiologies of pathogenesis of SARS-CoV-2 in COVID-19. Thirty-seven identified proteins in SARS-CoV-2 were evaluated.

## 2 Methods

The investigative data analysis procedure included the following steps. First, for each ORF (open reading frame) reported as a canonical CDS (coding sequence) for the SARS-CoV-2 virus, flagged (significant) immunogenetic SARS-CoV-2 epitopes were found using SVMTriP (http://sysbio.unl.edu/SVMTriP; [4]. These immunogenetic epitopes were then compared to human proteins using p-Blast (settings optimized for short sequences) against the *Homo sapiens* entries in the Protein Databank (https://blast.ncbi.nlm.nih.gov/). A list of human peptides with high local homology was compiled and their roles in the pathogenesis of COVID-19 from SARS-CoV-2 infection noted. The protein entries were mapped to nucleotide accession number, which were used to map as a gene set to pathways via Reactome (Reactome.org). Tissue distribution of the targeted proteins was explored using the Protein Atlas (ProteinAtlas.org).

## 3 Results

Thirty-seven SARS-CoV-2 proteins were downloaded from the NCBI SARS-CoV-2 NCBI resource. Of these, 8 proteins had no recognizably immunogenic peptides. The remaining proteins had between one and six immunogenic peptides (Table 1). The proteins with the largest number of immunogenic peptides were the Spike, or S protein (N ​= ​6 in total), and the non-structural protein NS3 (also N ​= ​6; Table 1). All of the proteins had at least one match to human proteins except one, specifically nucleocapsid phosphoprotein (epitope ‘QQQQGQTVTKKSAAEASKKP’), however even nucleocapsid phosphoprotein has one other epitope (‘RRGPEQTQGNFGDQELIRQG’) which has a localized match to the immunoglobulin heavy chain junction region (MOO20493; GNFGDQ).

**Table 1:** 

Protein	Accession	SARS-CoV-2 Protein Name	Immunogenetic epitope(s)	Accession	Human Protein Name	Putative pathogenic priming peptide (self-antigen)	Tissue RNA/Protein Expression
1	QHN73821	ORF1ab polyprotein	RARTVAGVSICSTMTNRQFH	CCO13833	alternative protein TJP1	TVICDTMLCPKVYFFTNRQF	nearly ubiquitous
		(partial)	CSTMTSR	MCD32611	**IHCJ**	CSTMTSR	B-cells, plasma cells
2	QHN73794	ORF1ab protein	VATLQAENVTGLFKDCSKVI	NP_001339255	la-related protein 4 (LARP4)	EEVKGLFKSENCPKVI	ubiquitous
3	QIA98594	ORF1ab polyprotein	DRRATCFSTASDTYACWHHS	AIT38911	**IGJHCVR**	DTYACW	B-cells, plasma cells
			DRRATCFSTASDTYACWHHS	AGK29065	**IGLCVR**	DTYACW	B-cells, plasma cells
			DRRATCFSTASDTYACWHHS	NP_001712	cytoplasmic tyrosine-protein kinase BMX isoform 1	ASDTYACWH	epididymous
4	QIA98597	ORF3a protein	YQIGGYTEKWESGVKDCVVL	AAH82244	PH domain and leucine rich repeat protein phosphatase 1(PHLPP1)	GYTEASGVKNKLCV	brain, lung, kidney
5	QIA98604	ORF7a protein	GVKHVYQLRARSVSPKLFIR	XP_016871107	sortilin related VPS10 domain containing receptor 1(SORCS1)	GIKHVYQ	brain, gastrointestinal tract, kidney
6	QIA98601	ORF8 protein	FYSKWYIRVGARKSAPLIEL	EAX03800	ankyrin repeat and sterile alpha motif domain containing 1A (ANKS1A)	RVGVRKSAVPL	eye, lung, gastrointestinal tract, liver
7	YP_009725310	endoRNAse	LIGEAVKTQFNYYKKVDGVV	3SWR_A	DNA methyltransferase 1	VGEAVKTDGKKSYYKKV	brain, lung, gastrointestinal tract
8	YP_009725308	helicase	ATNYDLSVVNARLRAKHYVY	MOQ41699	**IHCJ**	SVVAARLRPSHFDY	B-cells, plasma cells
9	YP_009725297	leader protein	LPQLEQPYVFIKRSDARTAP	5SZF_L	**Chain L, 2A10 antibody FAB fragment light chain**	PYVFGGGTKLEIKRADAAP	
			LPQLEQPYVFIKRSDARTAP	3QXM_A	glutamate ionotropic receptor kainate type subunit 2 (GRIK2)	LEEPYVLFKKSD	brain, kidney
10	QHO62114	matrix protein	FIASFRLFARTRSMWSFNPE	MCD74337	**IHCJ**	ARERSGWSFDP	B-cells, plasma cells
11	QIA98599	membrane glycoprotein	FIASFRLFARTRSMWSFNPE	MCD74337	**IHCJ**	ARERSGWSFDP	B-cells, plasma cells
12	QIH45026	M protein	FIASFRLFARTRSMWSFNPE	MCD74337	**IHCJ**	ARERSGWSFDP	B-cells, plasma cells
			FIASFRLFARTRSMWSFNPE	AAH80580	pentatricopeptide repeat domain 1(PTCD1)	RLFARARPM	ubiquitous
13	YP_009725298	nonstructural protein NS2	DGISQYSLRLIDAMMFTSDL	NP_001165883	VANGL planar cell polarity protein 1(VANGL1)	GIVQYAVSLVDALLF	ubiquitous
14	YP_009725298	nonstructural protein NS2	VEKKKLDGFMGRIRSVYPVA	EAW65335	adaptor protein containing pH domain, PTB domain and leucine zipper motif 1, isoform CRA_a	LVDAMMF	
15	YP_009725299	nonstructural protein NS3	LGYVTHGLNLEEAARYMRSL	AAC64695	supervillin (SVIL)	VTHRLLEEDTPRYMR	ubiquious except eye, blood
			EEVGHTDLMAAYVDNSSLTI	XP_016864345	Rap guanine nucleotide exchange factor 2 (RAPGEF2)	MASYVDNS	brain, bone marrow (RNA)
			EEVGHTDLMAAYVDNSSLTI	XP_016864345	Rap guanine nucleotide exchange factor 2 (RAPGEF2)	ESSSLT	brain, bone marrow (RNA)
			QTTLKGVEAVMYMGTLSYEQ	ANO56871	**T-cell receptor beta chain variable region**	MYLCASSLSYEQ	lung, bone marrow, blood
			QTTLKGVEAVMYMGTLSYEQ	NP_001292017	*N*-acetyltransferase 9 isoform	GTEAVLAM--LSYE	spleen
			QVESDDYIATNGPLKVGGSC	CAA56042	protein-tyrosine-phosphatase	DYIATQGPLK	ubiquitous
16	YP_009725300	nonstructural protein NS4	VHVMSKHTDFSSEIIGYKAI	AIT39025	**IGJHCVR**	THFNSEIIGY	B-cells, plasma cells
			VHVMSKHTDFSSEIIGYKAI	2W3C_AL	General vesicular transport factor P115	IHVLQTDRSDSEIIGY	skeletal muscle, others
17	YP_009725303	nonstructural protein NS7	GAVDINKLCEEMLDNRATLQ	5VHJ_D	Proteasome 26S subunit, ATPase 4(PSMC4)	GADINSICQESGMLAVRENR	ubiquitous
18	YP_009725304	nonstructural protein NS8	AVANGDSEVVLKKLKKSLNV	AAI11491	Bromodomain and WD repeat domain containing 3(BRWD3)	VANGDGEVV	ubiquitous
19	YP_009725305	nonstructural protein NS9	AKVTSAMQTMLFTMLRKLDN	1A4P_A	S100 calcium binding protein A10(S100A10)	AMETMMFT	lung, blood
			AKVTSAMQTMLFTMLRKLDN	P0CL83	Antigen 3-like protein 1	MIFSMLRKL	
20	YP_009725306	nonstructural protein NS10	TLKNTVCTVCGMWKGYGCSC	EAW66814	hCG1795641	KGYGCSC	
			TLKNTVCTVCGMWKGYGCSC	EAW84736	Cartilage oligomeric matrix protein	LKNTVMECDACGM	adipose tissue, muscle
21	YP_009725307	RNA-dependent RNA polymerase	QYIRKLHDELTGHMLDMYSV	NP_001271153	Elongator acetyltransferase complex subunit 3 (ELP3)	FIRNLHDALSGH	nearly ubiquitous
			MPNMLRIMASLVLARKHTTC	XP_011508049	Hedgehog acyltransferase (HHAT)	MATLLARKH	nearly ubiquitous
			DVNLHSSRLSFKELLVYAAD	XP_006713353	Semaphorin 3F(SEMA3F)	RLSFKEL	nearly ubiquitous
22	QIH45023	S protein	LNEVAKNLNESLIDLQELGK	EAW69281	hCG23535	KNLNQSLLDLHALG	
			TLVKQLSSNFGAISSVLNDI	XP_016871528	Attractin-like protein 1	FGAISSVLNDI	brain
			TLVKQLSSNFGAISSVLNDI	XP_016871528	Attractin-like protein 1	AIASALIDI	brain
			QQLIRAAEIRASANLAATKM	XP_011528323	tetratricopeptide repeat protein 28	QQLGIAEDLKDRAAEGRASSN	ubiquitous
			KEELDKYFKNHTSPDVDLGD	XP_024309095	follistatin-related protein	EILDKYFKN	placenta, most others
			VMVTIMLCCMTSCCSCLKGC	AAO32957	Metallothionein 1E (MT1E)	CKTSCCSC	liver, most others
23	QIA98596	Spike protein	LNEVAKNLNESLIDLQELGK	XP_011535432	Coiled-coil domain-containing protein 175 isoform X8	KNMEEGLITLQEL	brain, pituitary gland, testis
			TLVKQLSSNFGAISSVLNDI	AAH27241	ALDH1L1 protein	LVKNIQLEDGKMILASNFFKGAASSVL	
			QQLIRAAEIRASANLAATKM	XP_011528323	Tetratricopeptide repeat protein 28 isoform X8	QQLGIAEDLKDRAAEGRASSNL	ubiquitous
			KEELDKYFKNHTSPDVDLGD	XP_024309095	Follistatin-related protein 1 isoform X1	EILDKYFKN	placenta, most others
			VMVTIMLCCMTSCCSCLKGC	NP_149050	Keratin associated protein 4-7(KRTAP4-7)	CCMSSCC	skin
24	YP_009725301	3C-like proteinase	AENVTGLFKDCSKVITGLHP	AAH22377	La ribonucleoprotein domain family member 4 (LARP4)	EEVKGLFKSENCPKVI	ubiquitous
			HLSVDTKFKTEGLCVDIPGI	XP_024308868	Titin	DTKFKTTGLDEGL	heart muscle, skeletal muscle
25	QIA98602	nucleocapsid phosphoprotein	RRGPEQTQGNFGDQELIRQG	MOO20493	**IHCJ**	GNFGDQ	B-cells, plasma cells
26	QHR63265	nonstructural protein NS7a	GVKHVYQLRARSVSPKLFIR	XP_016871107	VPS10 domain-containing receptor SorCS1	GIKHVYQ	thyroid gland, many others
27	QHR63267	nonstructural protein NS8	FYSKWYIRVGARKSAPLIEL	AAH31934	Ankyrin repeat and sterile alpha motif domain containing 1A	RVGVRKSAVPL	ubiquitous
28	QIA98602	nucleocapsid phosphoprotein	QQQQGQTVTKKSAAEASKKP	n/a	n/a	n/a	
29	QIA98603	ORF10 protein	MGYINVFAFPFTIYSLL- LCRMNSRNYIAQVDVVNFNLT	n/a	n/a	n/a	
30	QHR63254	nonstructural protein NS6	n/a	n/a	n/a	n/a	
31	QHR63276	nonstructural protein NS7b	n/a	n/a	n/a	n/a	
32	QHW06053	orf6 protein	n/a	n/a	n/a	n/a	
33	QIA20050	ORF7b protein	n/a	n/a	n/a	n/a	
34	YP_009725312	nsp11	n/a	n/a	n/a	n/a	
35	YP_009725311	2′-O-ribose methyltransferase	n/a	n/a	n/a	n/a	
36	YP_009725309	3′-to-5′ exonuclease	n/a	n/a	n/a	n/a	
37	QIH45025	E protein	n/a	n/a	n/a	n/a	

immunoglobulin heavy chain junction region							

Remarkably, over 1/3 (11/27) of the immunogenic proteins in SARS-CoV-2 have potentially problematic homology to proteins that are key to the human adaptive immune system (emboldened in Table 1). Mapping of the overall gene list to Pathways via Reactome.org revealed that many functions of the human adaptive immune system might be impacted via autoimmunity against these proteins and their interactors, including MCH Class I and Class II antigen presentation, PD-1 signaling, cross-presentation of soluble exogenous antigens and the ER-Phagosome pathway.

## 4 Discussion

These results could explain in part the high rates of serious illness associated with SARS-CoV-2. They could also explain the lengthy asymptomatic period prior to presentation of symptoms characteristic of COVID-19. SARS-CoV-2 could impair the immune response, at first, and then, over time, the immune system could begin to mount an attack on the myriad of proteins. Most of the identified human target proteins had low overall homology but high local homology over short segments of their epitopes. The Protein Atlas results indicated that numerous proteins are expressed in a variety of tissues as noted in Table 1.

Unintended consequences of pathogenesis from vaccines are not new, nor are they unexpected. They are unanticipated only if those who develop them do not include available knowledge in their formulation plan. For example, the H1N1 influenza vaccine used in Europe led to narcolepsy in some patients, resulting from homology between the human hypocretin (aka, orexin) receptor 2 molecule and proteins present in the vaccine. This was established via the detection of cross-reactive antibodies in the serum of patients who develop narcolepsy following H1N1 vaccination in Europe [5].

The fact that pathogenic priming may be occurring involving autoimmunity against multiple proteins following CoV vaccination is consistent with other observations observed during autoimmunity, including the release of proinflammatory cytokines and cytokine storm. Similar to the SARS-CoV animal studies [6], found that mice vaccinated against MERS-CoV (Middle East Respiratory Syndrome) development exaggerated pulmonary immunopathology when challenged with the MERS virus following vaccination. They reported that lung mononuclear infiltrates were observed in all groups after virus challenge, and that increased infiltrates that contained eosinophils and the eosinophil promoting IL-5 and IL-13 cytokines were observed only in the vaccinated animals.

Pathogenic priming may be more or less severe in vaccine or infection induced immune responses to some proteins than for others due to original antigenic sin; the immunologic reaction against self-antigens may be made less severe as fast-evolving viruses evolve away from the original vaccine type. Thus, the screening of immunogenic epitopes for pathogenic priming potential via homology may be augmented by studies of autoantibodies that cross-react with epitopes included in vaccines.

SARS-CoV-2 has some unexplained pathogenic features that might be related to the table of putative pathogenic priming peptides. Exposure to these specific peptides - via either infection or vaccination - might prime patients for increased risk of enhanced pathogenicity during future exposure due either to future pandemic or outbreaks or via universal vaccination programs. While the mechanisms pathogenesis of COVID-19 are still poorly understood, the morbidity and mortality of SARS has been extensively studied. Thus, the involvement of pathogenic priming in re-infection by COVID-19 is a theoretical possibility; of course no vaccine against SARS-CoV-2 has yet been tested in animals and therefore we do not yet know if pathogenic priming is in fact expected. Such studies should be undertaken before use of any vaccine against SARS-CoV-2 is used in humans.

## Declaration of competing interest

Dr. Lyons-Weiler has, in the past, served as expert witness in the National Vaccine Injury Compensation Program.

## Statement of interests

The author has served as an expert witness in the US National Vaccine Injury Compensation Program.
